# Supplementary material for: Moderate levels of 5-fluorocytosine cause the emergence of high frequency resistance in cryptococci
Source: Nat Commun. 2021 Jun 8;12:3418. doi: 10.1038/s41467-021-23745-1 (PMC8187385; doi:10.1038/s41467-021-23745-1)
Supplement: Supplementary file 1 — Supplementary Information [file 41467_2021_23745_MOESM1_ESM.pdf]

Supplementary Information for

Moderate levels of 5-fluorocytosine cause the emergence of high frequency resistance in cryptococci

Yun C. Chang[1*] Ami Khanal Lamichhane[1], Hongyi Cai[2], Peter J. Walter[2], John E. Bennett[3], Kyung J. Kwon-Chung[1]

*Corresponding author: E-mail: ychang@niaid.nih.gov

Description of Supplementary Files

Supplementary Fig. 1. 5-FC resistance colonies emerge at high frequency at low concentrations of 5-FC. Cells of each indicated strain were plated on YNB media containing 5-FC. The concentration of 5-FC was approximately 20-, 40-, 100-, and 400-fold MIC of each strain as indicated. The number of cells plated on each plate are specified. Plates were incubated at 30°C for 7 days and photographed.

Supplementary Fig. 2. The stability of 5-FC resistant clones. Fifteen each of large- and small-sized 5-FC resistant clones of each strain were daily transferred in YPD media and the percentage of the 5-FC resistant population was determined periodically. Each clone is represented by a different color and symbol as indicated at the bottom of the figure. The "L" and "S" respectively indicates the large and small size colony at the time of its isolation. The nature of variations in some of the clones were not clear.

Supplementary Table 1. List of strains relevant to the study.

Supplementary Table 2. List of primers used in the study

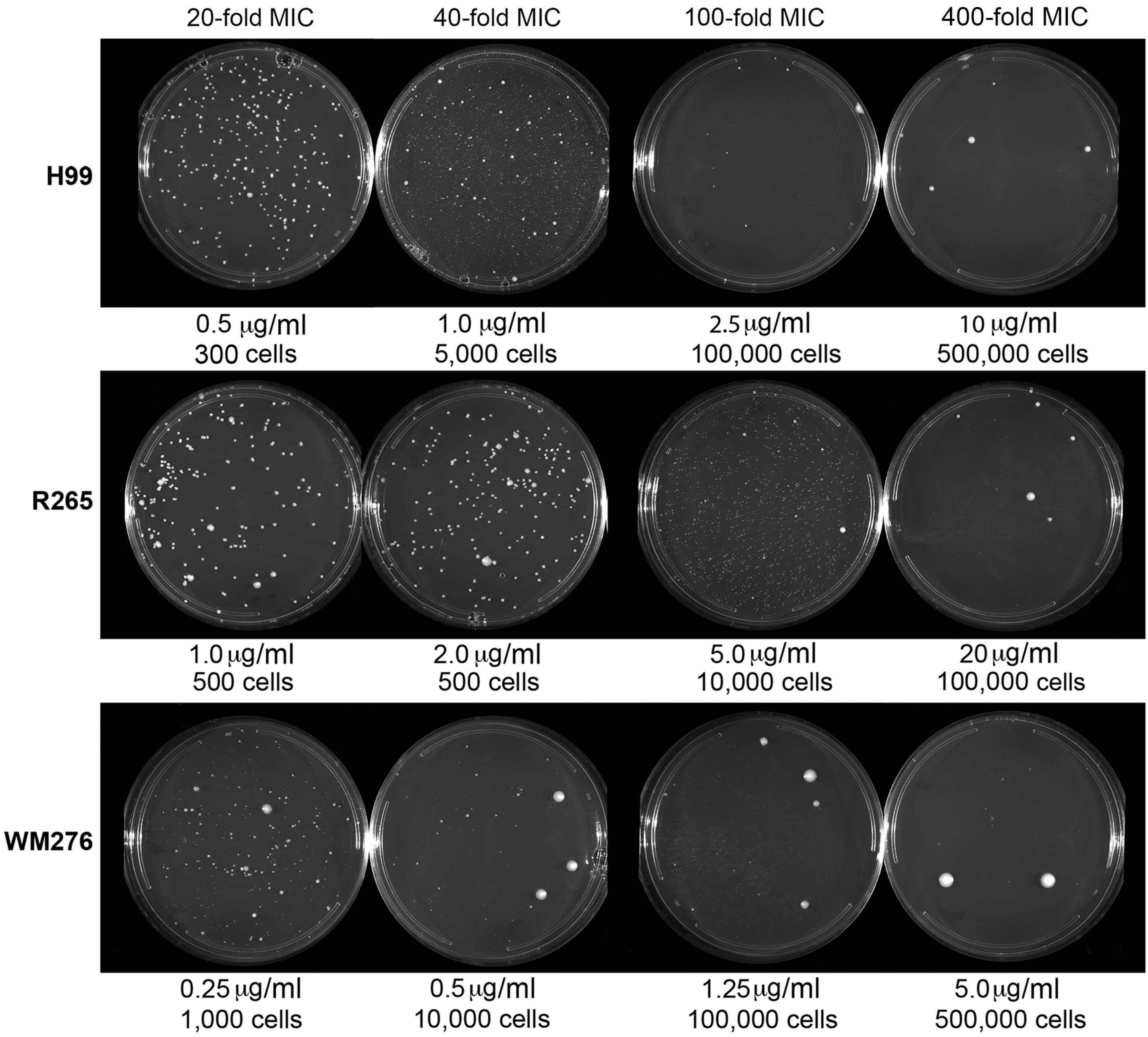

Supplementary Fig. 1. 5-FC resistance colonies emerge at high frequency at low concentrations of 5-FC. Cells of each indicated strain were plated on YNB media containing 5-FC. The concentration of 5-FC was approximately 20-, 40-, 100-, and 400-fold MIC of each strain as indicated. The number of cells plated on each plate are specified. Plates were incubated at 30°C for 7 days and photographed.

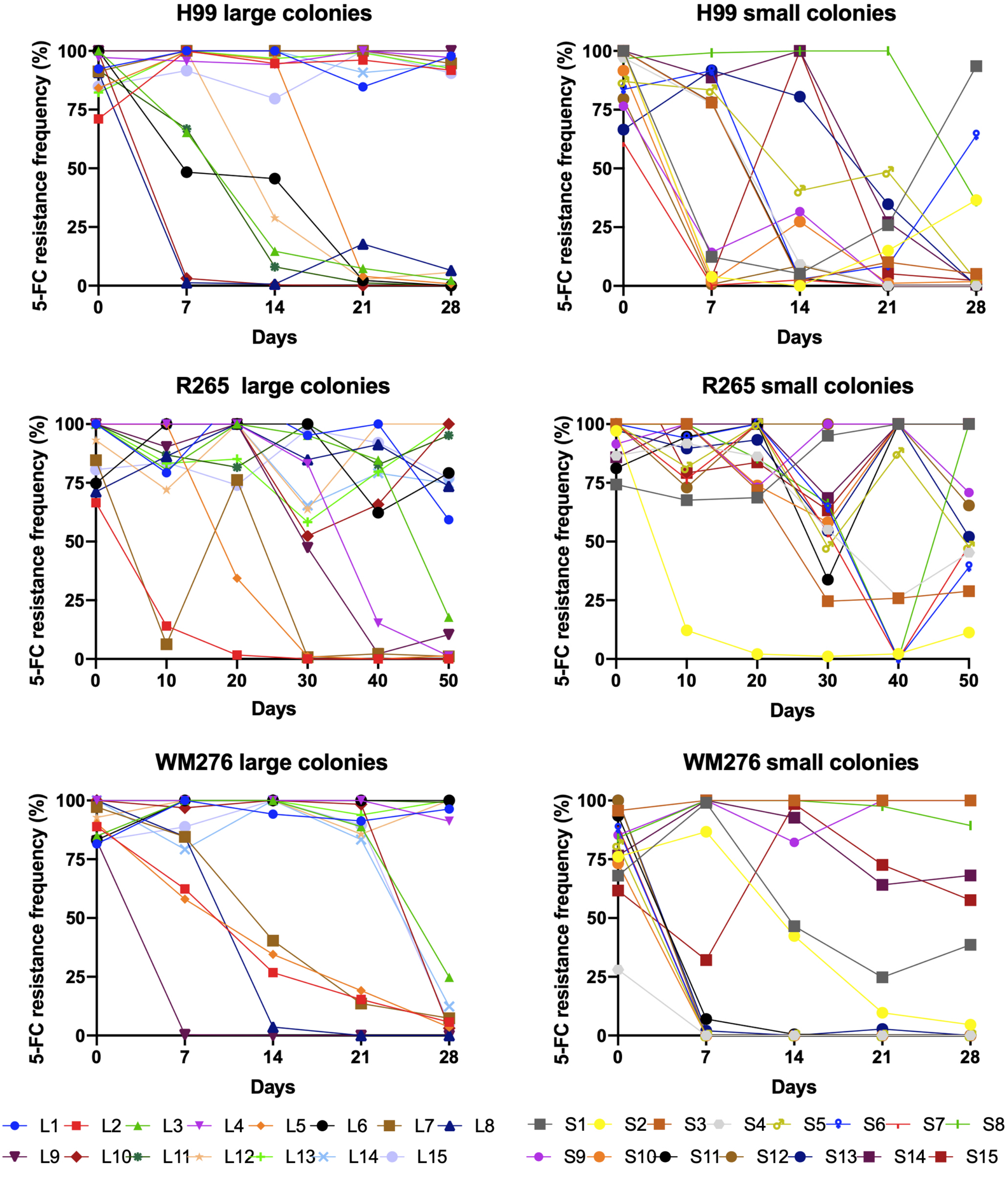

Supplementary Fig. 2. The stability of 5-FC resistant clones. Fifteen each of large- and small-sized 5-FC resistant clones of each strain were daily transferred in YPD media and the percentage of the 5-FC resistant population was determined periodically. Each clone is represented by a different color and symbol as indicated at the bottom of the figure. The “L” and “S” respectively indicates the large and small size colony at the time of its isolation. The nature of variations in some of the clones were not clear.

Supplementary Table 1. List of strains relevant to the study

Strains	Genotype	Strain description
H99 related strains		
H99	wild type	*C. neoformans* VNI
C1918	*fcy1Δ::NEO*	*fcy1* deletant
C1931	*fcy2Δ::HYG*	*fcy2* deletant
C1920	*fur1Δ::NEO*	*fur1* deletant
KN99α	wild type	Isogenic strain derived from H99
13C2	*uxs1Δ::NAT*	From FGSC, deposited by H. Madhani
C1952	*uxs1Δ::NAT*	47 transfers of 13C2
C2050	*uxs1Δ::NAT, nrg1::NEO*	*Knrg1* in 13C2
C2119	*uxs1Δ::NAT,* UGD^{G19A}*::NEO*	*Kugd1* in 13C2
C2133	*nrg1::NEO*	*Knrg1* in KN99α
C2168	UGD^{G19A}*::NEO*	*Kugd1* in KN99α
32C12	CNAG_04784 deletant	From FGSC, deposited by H. Madhani
12B11	*ugd1Δ::NAT*	From FGSC, deposited by H. Madhani
41H10	*nrg1Δ::NAT*	From FGSC, deposited by H. Madhani
R265 related strains		
R265	wild type	*C. gattii* VGIIa
C1960	*fcy1Δ::NEO*	*fcy1* deletant
C1911	*fcy2Δ::HYG*	*fcy2* deletant
C2178	*fur1Δ::NEO*	*fur1* deletant
C2172	*CNBG_2198Δ::HYG*	CNBG_2198 deletant
WM276 related strains		
WM276	wild type	*C. gattii* VGI
C2053	*uxs1Δ::HYG*	*uxs1* deletant
C2074	*uxs1Δ::HYG UXS1::NEO*	*uxs1Δ* complemented strain
C2104	*uxs1Δ::HYG,* UGD^{G19A}*::NAT*	*Wugd1* in C2053
C2061	UGD^{G19A}*::NAT*	*Wugd1* in WM276
C2067	*nrg1::NEO*	*Wnrg1* in WM276
C2072	*uxs1Δ::HYG, nrg1::NEO*	*Wnrg1* in C2053
C2113	*uxs1Δ::HYG, Xnrg1::NEO*	*Xnrg1* in C2053
C1832	*fcy1Δ::HYG*	*fcy1* deletant
C1384	*fcy2Δ::HYG*	*fcy2* deletant
C1900	*fur1Δ::NEO*	*fur1* deletant
C2022	*fur1Δ::NEO*	44th transfers of C1900
C2080	$URA6^{V172L}$*::NEO*	$URA6^{V172L}$
C2171	$DPB2^{G508C}$*::NEO*	$DPB2^{G508C}$

WL8 derived strains		
WL8	*uxs1*	derived from WM276
C1933	*uxs1*	51 transfers of WL8
C2143	*uxs1, UGD^{G19A}::NAT*	*Wugd1* in WL8
C2028	*uxs1, BCK1^{R1382*}::NEO,*	*BCK1^{R1382}* in WL8
WL9 derived strains		
WL9	*uxs1*	derived from WM276
C1943	*uxs1*	WL9 51 transfers
C2030	*uxs1, UGD^{G19A}::NAT*	*Wugd1* in WL9
C2029	*uxs1, RAN1^{Y45*}::HYG*	*RAN1^{Y45*}* in WL9
C2071	*uxs1*, *nrg1::NEO*	*Wnrg1* in WL9
C2111	*uxs1*, *Xnrg1::NEO*	*Xnrg1* in WL9
copy number related		
C1976	heteroresistant to fluconazole	derived from H99
C1977	heteroresistant to fluconazole	derived from H99
C2047	17 transfers of C1976	this study
C2048	17 transfers of C1977	this study
C1881	*AFR1, AFR1::NEO*	2 copies of *AFR1* in H99
C2055	*AFR1, AFR1::NEO*	≥2 copies of *AFR1* in R265
C2056	*AFR1, AFR1::NEO*	≥2 copies of *AFR1* in R265
C2057	*AFR1, AFR1::NEO*	≥2 copies of *AFR1* in WM276
C2058	*AFR1, AFR1::NEO*	≥2 copies of *AFR1* in WM276
FCY2 tagged strains		
C2151	*UXS1, FCY2-mNG-FLAG::NEO*	derived from WM276
C2152	*uxs1Δ::HYG, FCY2-mNG-FLAG::NEO*	derived from C2053
C2153	*uxs1Δ::HYG, UGD^{G19A}::NAT, FCY2-mNG-FLAG::NEO*	derived from C2101

Supplementary Table 2. List of primers used in the study

Gene	primer	Sequence
H99 related		
Fcy1_CNAG_00613	Hfcy1A	CCCATTCAAGCTCCTCCATA
	Hfcy1B	GCTAGTTTCTACATCTCTTCCGTGGTTGCTATTGGGCCAAGAAA
	Hfcy1C	**CGCC**GCTCTCCAGCTCACATCCTCCGTGATCAACCTCGATTGTG
	Hfcy1D	AGCTCAGCGATTGGGTAGAA
Fcy2_CNAG_01681	Hfcy2A	GACCTGGAGGCACTGATGAT
	Hfcy2B	GCTAGTTTCTACATCTCTTCCGTGCAACAGGCTCAAATCCCTTC
	Hfcy2C	**CGCC**GCTCTCCAGCTCACATCCTCCCGATAAGGTCTGCCAGCTA
	Hfcy2D	TTAGCAGCTTTCCTCGGCTA
Fur1_CNAG_02337	Hfur1A	CAAGGCTGCCAGGAAAATTA
	Hfur1B	GCTAGTTTCTACATCTCTTCCGTGTGCAAACAGCAAAACTGGAG
	Hfur1C	**CGCC**GCTCTCCAGCTCACATCCTCACAGGTGGGTGCTATGCTCT
	Hfur1D	TACGACCGCAGTTGTAGCAG
Nrg1 in KN99	Nrg1A	TTGGTACCGAGCTCGGATCCCTCAACCATCAAAGGGGAAA
Knrg1	Nrg1B	AAGACACATAAAGCACCCGAGGCTGGGC
	Nrg1C	TCGGGTGCTTTATGTGTCTTTACCCATATCATTAGGC
	Nrg1D	CACTGGCGGCCGTTACTAGTACCCCCGCAGATAGAGAAAT
	Nrg1E	AAGGGCGAATTCTGCAGATCTCTATCTGCGGGGGTACAA
	Nrg1F	CCTCTAGATGCATGCTCGAGAGGGGAGAGTGCTTGGAAAT
Ugd1_CKF_04969	Ugd1A2	TTGGTACCGAGCTCGGATCCCCATTAGCACCTGCCATTTT
G19A	Ugd1B2	CACTGGCGGCCGTTACTAGTTCGCCAGTTCCATCTTCTCT
	Ugd1C2	AAGGGCGAATTCTGCAGATTTGCTATGTGGCGGGTCTAT
	Ugd1D2	CAAGTGGGGGCGCCTGGAAGat
	Ugd1E2	ATCTTCCAGGCGCCCCCACTTG (Gly to Ala)
	Ugd1F2	CCTCTAGATGCATGCTCGAGCAAGAACCACCGAAACCCTA
UGD1_qRT-PCR	Ugd1Fr	GCTGTATCGGCGCTGGTT
	Ugd1Rv	GGGCACTTCAACGCGATAAC
R265 related		
Fcy1_CNBG_1313	RFCY1A	CCCATTCAAGCTCCTCCATA
	RFCY1B	GCTAGTTTCTACATCTCTTCCGTGAGGAAACAGAGTCGCGTGAT
	RFCY1C	**CGCC**GCTCTCCAGCTCACATCCTCAGTCCTGGCGGTAAAGTGTG
	RFCY1D	GAAGCTCAGCGATTGGGTAG
Fcy2_CNBG_3227	RFCY2A	AAGGCCTATTCAGCGACAGA
	RFCY2B	GCTAGTTTCTACATCTCTTCCGTGCCTTGGGATCATCAGGCTTA
	RFCY2C	**CGCC**GCTCTCCAGCTCACATCCTCGATAAGGCGTGCCAACTAT
	RFCY2D	CTGAATCTCGGAAAGGCTTG
Fur1_CGB_E2660C	Fur1A	CTCAGCGATAGTGGGGAGAG
	Fur1B	GCTAGTTTCTACATCTCTTCCGTGAGTCGTGATGTTGGTGGACA
	Fur1C	**CGCC**GCTCTCCAGCTCACATCCTCGTGGGTGCCATGCTCTTACT
	Fur1D	GCAAACCGAATCGAAATTGT
Fcy2 tag	Fcy2TA2	ACCATGATTACGCCAAGCTTATGGGTCGCATTCTGGTAAG
	Fcy2TB2	CACTGGCGGCCGTTACTAGTTCTTGTTCTGCCCTCTCGAT
	Fcy2TC2	AAGGGCGAATTCTGCAGATGATACTGGCCCCCGTAAAGT
	Fcy2TD2	TCCTCACCCTTCGATACCATGTTGAGTCTTAAAGTGTCGAGATATAT
	mNG1	ATGGTATCGAAGGGTGAGGA
	mNG4	CTTATCGTCGTCGTCTTTGTAGTC
	Fcy2TE	GACTACAAAGACGACGACGATAAGATGTCAGATATCGAGAAGGCAG
	Fcy2TF	CCTCTAGATGCATGCTCGAGCCATCGTAAAACCCCATACG
WM276 related		
Fcy1_CGB_A6420C	FCY1A	GCGATGGGAGAGTAAATCCA
	FCY1B	GCTAGTTTCTACATCTCTTCCGTGGGTTTAGCTGGGGATCCTTC
	FCY1C	**CGCC**GCTCTCCAGCTCACATCCTCAGTCCTGCCGGTAAAGTGTG
	FCY1D	CCTCTCCAAGGTCGGTGATA
Fcy2_CGB_C2750W	FCY2A	AACCTGCTTCTAACGCGAAA
	FCY2B	GCTAGTTTCTACATCTCTTCCGTGCCTTGGGATCATCAGGCTTA
	FCY2C	**CGCC**GCTCTCCAGCTCACATCCTCCTTATCCTGCCTTCCGAGTG

	FCY2D	GAAGCTTGCGGACATGAAGT
Fur1_CGB_E2660C	Fur1A	CTCAGCGATAGTGGGGAGAG
	Fur1B	GCTAGTTTCTACATCTCTTCCGTGAGTCGTGATGTTGGTGGACA
	Fur1C	**CGCC**GCTCTCCAGCTCACATCCTCGTGGGTGCCATGCTCTTACT
	Fur1D	GCAAACCGAATCGAAATTGT
Uxs1_CGB_G3260C	Uxs1A	ATTTGCGTCAGCATCCCTAC
	Uxs1B	GCTAGTTTCTACATCTCTTCCGTGGAAGGGTGTGGATGGGTATG
	Uxs1C	**CGCC**GCTCTCCAGCTCACATCCTCTGTGCATTTTGCCAATTTGT
	Uxs1D	ACATCCACACCTCGTTCCTC
Uxs1 complementation	Uxs1G	TTGGTACCGAGCTCGGATCCTGCGCGCATTTCTTATACTG
	Uxs1H	TACATCTCTTCCGTGGCGCGCCGTAGGGGTCAAATTCCGACA
Nrg1_CGB_J1350C	Nrg1A2	TTGGTACCGAGCTCGGATCCACCATCTCAGCCATCAAAGG
Wnrg1	Nrg1B2	TAGACATGTAACGCACCAGAGGTGGGGC
	Nrg1C2	TCTGGTGCGTTACATGTCTATATATCCCCATTAGTAGC
	Nrg1D2	CACTGGCGGCCGTTACTAGTTTTTTATGGGCTGCGTTGTT
	Nrg1E2	AAGGGCGAATTCTGCAGATAGCGCCAATCTTGTCTCAGT
	Nrg1F2	CCTCTAGATGCATGCTCGAGGCCACGCAGATAACGAAAA
Bck1_CGB_I2500W	BCK1A	TTGGTACCGAGCTCGGATCCCCATAACACAAGCCCTCGAT
R1382*	BCK1B	GTCTTCGTGTCAGGAGATTTGAC
	BCK1C	GTCAAATCTCCTGACACGAAGAC
	BCK1D	CACTGGCGGCCGTTACTAGTGATGTCATGCGCCCTACTTT
	BCK1E	AAGGGCGAATTCTGCAGATTGTGCATTATGCGATGGACT
	BCK1F	CCTCTAGATGCATGCTCGAGTTGGCTCCTTACCAACCAAC
Ran1_CGB_F0330C	Ran1A	TTGGTACCGAGCTCGGATCCAAGCATCCGAACGAAGAGAA
Y45*	Ran1B	CACTGGCGGCCGTTACTAGTGGTTCGCTGGTTACCCTGTA
	Ran1C	AAGGGCGAATTCTGCAGATAATTTTGAGTCCGCAAGTCG
	Ran1D	GCTGCGGCTATCTTGAGTCAAC
	Ran1E	GTTGACTCAAGATAGCCGCAGC
	Ran1F	AGATGCATGCTCGAGCGGCCGCGACGTGGTAGGGGAAAGACA
Ugd1_CGB_D0330C	Ugd1A	TTGGTACCGAGCTCGGATCCGCTGGTCGGTAGGGAACATA
G19A	Ugd1B	CACTGGCGGCCGTTACTAGTAACCTGGAATGGCTTTGATG
	Ugd1C	AAGGGCGAATTCTGCAGATTGTGGCGATATTCAAATGG
	Ugd1D	CAAGTGGGAGCGCCTGGAGGAT
	Ugd1E	ATCCTCCAGGCGCTCCCACTTG
	Ugd1F	CCTCTAGATGCATGCTCGAGTAGGAACCCAGTTGGCGTAG
Ura6_CGB_F6195W	Ura6A	TTGGTACCGAGCTCGGATCCCCCTCTTGTGAACGTCGTG
V172L	Ura6B	AAGAACAGCAAAAAGCTGGATTTA
	Ura6C	ATCCAGCTTTTTGCTGTTCTTTAG
	Ura6D	CACTGGCGGCCGTTACTAGTAGGACGAAATTCAAGGCGTA
	Ura6E	AAGGGCGAATTCTGCAGATCCCGCGAATGAGTAAAGAAG
	Ura6F	CCTCTAGATGCATGCTCGAGACAAGGGTGTCGGCATCTAC
CNBG_2198	Pcg1A	TCGCCCGACTACTTCCATAC
	Pcg1B	GCTAGTTTCTACATCTCTTCCGTGGACATTTTGGTGGGGACAAG
	Pcg1C	**CGCC**GCTCTCCAGCTCACATCCTCCAAGGCTTTCCAAGCGTAAA
	Pcg1D	ACGCTCAACACAACTGCAAG
Dpb2_CGB_F1400C	Dpb2A	TTGGTACCGAGCTCGGATCCACCCTCCGAGTGAAAGAAG
G508C	Dpb2B	AACAAACTTGCAAGGGTTAAAAACGTG
	Dpb2C	TTTTTAACCCTTGCAAGTTTGTTGGG
	Dpb2D	CACTGGCGGCCGTTACTAGTATCCTCCAAAGTGCTGACG
	Dpb2E	AAGGGCGAATTCTGCAGATACGTCAGCACTTTGGAGGAT
	Dpb2F	GGCGAATTGGGCCCTCTAGACGGAGCTTCTTGATGAGACC
copy number determination		
	chr1B probe	CTTATCGTCCGTATGCTGCGTTACCGC
	chr1B fr	TCGCCATCTACGCCTACGA
	chr1B rev	TGGCACGAGAGTGGCATCT
	chr1C probe	TGTCACAGCCCCTCCACCCACTTT
	chr1C fr	AATCCCTTCTTGCTCGTACTCTTC
	chr1C rev	GCCACATCCACTTCCTCCAA
