# Supplementary material for: Moderate levels of 5-fluorocytosine cause the emergence of high frequency resistance in cryptococci
Source: Nat Commun. 2021 Jun 8;12:3418. doi: 10.1038/s41467-021-23745-1 (PMC8187385; doi:10.1038/s41467-021-23745-1)
Supplement: Supplementary file 3 — Description of Additional Supplementary Files [file 41467_2021_23745_MOESM3_ESM.pdf]

Description of Additional Supplementary Files

File Name: Supplementary Data 1
Description: Variants detected in the 5-FC resistant clones derived from H99, R265 and WM276.

File Name: Supplementary Data 2
Description: Variants detected in suppressor strains derived from 13C2, WL8 and WL9.
